# Avian Influenza A(H5N1) Virus among Dairy Cattle, Texas, USA

**DOI:** 10.3201/eid3007.240717

**Published:** 2024-07

**Authors:** Judith U. Oguzie, Lyudmyla V. Marushchak, Ismaila Shittu, John A. Lednicky, Aaron L. Miller, Haiping Hao, Martha I. Nelson, Gregory C. Gray

**Affiliations:** University of Texas Medical Branch, Galveston, Texas, USA (J.U. Oguzie, L.V. Marushchak, I. Shittu, A.L. Miller, H. Hao, G.C. Gray);; University of Florida, Gainesville, Florida, USA (J.A. Lednicky);; National Center for Biotechnology Information, National Library of Medicine, National Institutes of Health, Bethesda, Maryland, USA (M.I. Nelson)

**Keywords:** influenza, viruses, cattle, influenza A virus, highly pathogenic avian influenza virus, epidemiology, Texas, United States, H5N1

## Abstract

During March and April 2024, we studied dairy cattle specimens from a single farm in Texas, USA, using multiple molecular, cell culture, and next-generation sequencing pathogen detection techniques. Here, we report evidence that highly pathogenic avian influenza A(H5N1) virus strains of clade 2.3.4.4b were the sole cause of this epizootic.

Since the arrival of clade 2.3.4.4b avian influenza A(H5N1) in North America in late 2021, frequent mammal spillover events have occurred in a diverse range of species, including 1 human infection, but those strains have not affected cattle. Cattle are known to be permissive but resilient to infection with influenza A, B, and C viruses (*1*); however, they are susceptible to influenza D virus, which is thought to have near-worldwide distribution (*2*). Influenza D virus is thought to move from cow-to-cow through direct contact or short-distance aerosol respiratory transmission (*2*), and possible occasional influenza D virus spillover to humans is a concern (*3*,*4*). Even so, influenza viruses are not the first pathogens veterinarians or veterinary diagnostic laboratories search for in studying cattle respiratory epizootics. We report results of an investigation into influenza virus infections among dairy cattle on a farm in Texas, USA.

## The Study

On March 18, 2024, we were notified of epidemics of illness among Texas dairy cattle. The cattle had transient respiratory and gastrointestinal signs (*5*). Veterinary diagnostic laboratory results were largely unremarkable except for rumors among cattle veterinarians of possible influenza A virus detection among cattle and conjunctivitis among dairy farm workers. The University of Texas Medical Branch (UTMB) research team offered diagnostic support owing to the team’s novel pathogen detection capabilities (*6*–*9*) and having recognized that conjunctivitis among workers handling animals had been previously noted in association with highly pathogenic avian influenza (HPAI) epizootics (*10*–*13*). On March 19, we were invited to investigate the outbreak by a farm owner. We provided the farm with sampling supplies and instructions. UTMB’s Institutional Animal Care and Use Committee has viewed such diagnostic work to be exempt from formal ethics review.

To determine the etiology of cattle illnesses, we used molecular screening and, in some cases, cell culture and metagenomics, to examine cattle swab specimens (Appendix 1). We targeted 6 viral groups, adenoviruses, coronaviruses, enteroviruses, influenza viruses, paramyxoviruses, and pneumoviruses, using previously published techniques (*8*).

At our request, on March 21, dairy farm management collected and shipped swab specimens from the nasal passages of 14 cows with signs of illness and 6 cows with no sign of illness in a shipping container with ice packs. We received the samples and completed questionnaires on March 22. To determine whether pathogens were enteric, we requested additional samples from the dairy farm on March 28. Nasal and rectal swab specimens were taken from 10 additional ill cows on April 1; we received those 20 additional swab specimens on April 3.

The 40 swab specimens were obtained from 30 different cows (24 sick and 6 healthy) from the same dairy farm (Appendix 1 Table 1); specific farm location, name, and cattle breed are withheld for privacy purposes. Sampled cattle ranged from 2 years 3 months of age to 7 years 10 months of age.

Farm staff first observed illnesses in cattle on March 6. Cattle with otherwise healthy records showed signs of decreased appetite, lethargy, increased respiratory secretions, high temperatures (up to 105°F or 40.56°C), abnormal bowel movements, and decreased milk production. During March 10–12, >4.75% of the herd had clinical signs of influenza-like illness and were being treated in the hospital pens. No dead birds, dead cats, or other deceased wildlife were observed. At the time of specimen collections, cattle illnesses were on the wane.

Several workers experienced influenza-like symptoms and missed work during March 4–6. A maternity worker visited a local clinic and received treatment for influenza-like symptoms; 2 milkers also experienced influenza-like symptoms and stayed home. No cases of conjunctivitis, severe illness, or hospitalizations were reported among workers.

**Figure 1:**
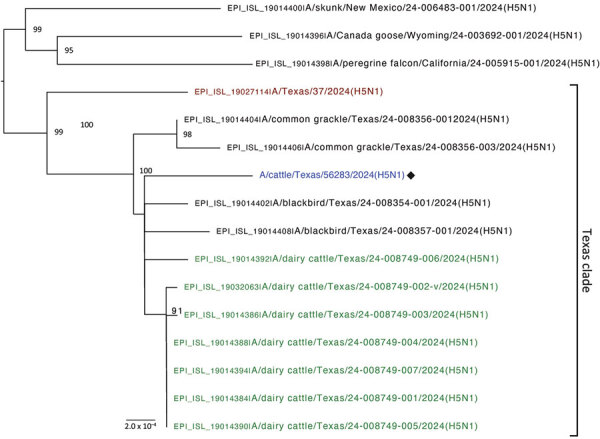
Phylogenetic tree of the concatenated genome in study of avian influenza A(H5N1) virus among dairy cattle, Texas, USA. Maximum-likelihood phylogenetic tree inferred for the A/cattle/Texas/5628356283/2024 (H5N1) virus isolated in this study (blue text) and 15 other closely related HPAI H5N1 viruses downloaded from GISAID (https://www.gisaid.org). Bootstrap values are provided for key nodes. The clade of 13 Texas viruses collected during March 2024 is labeled. Red text indicates human case (A/Texas/37/2024) and green text indicates cattle viruses collected from other farm(s) in Texas. Branch lengths are drawn to scale. Scale bar indicates number of substitutions per site.

**Figure 2:**
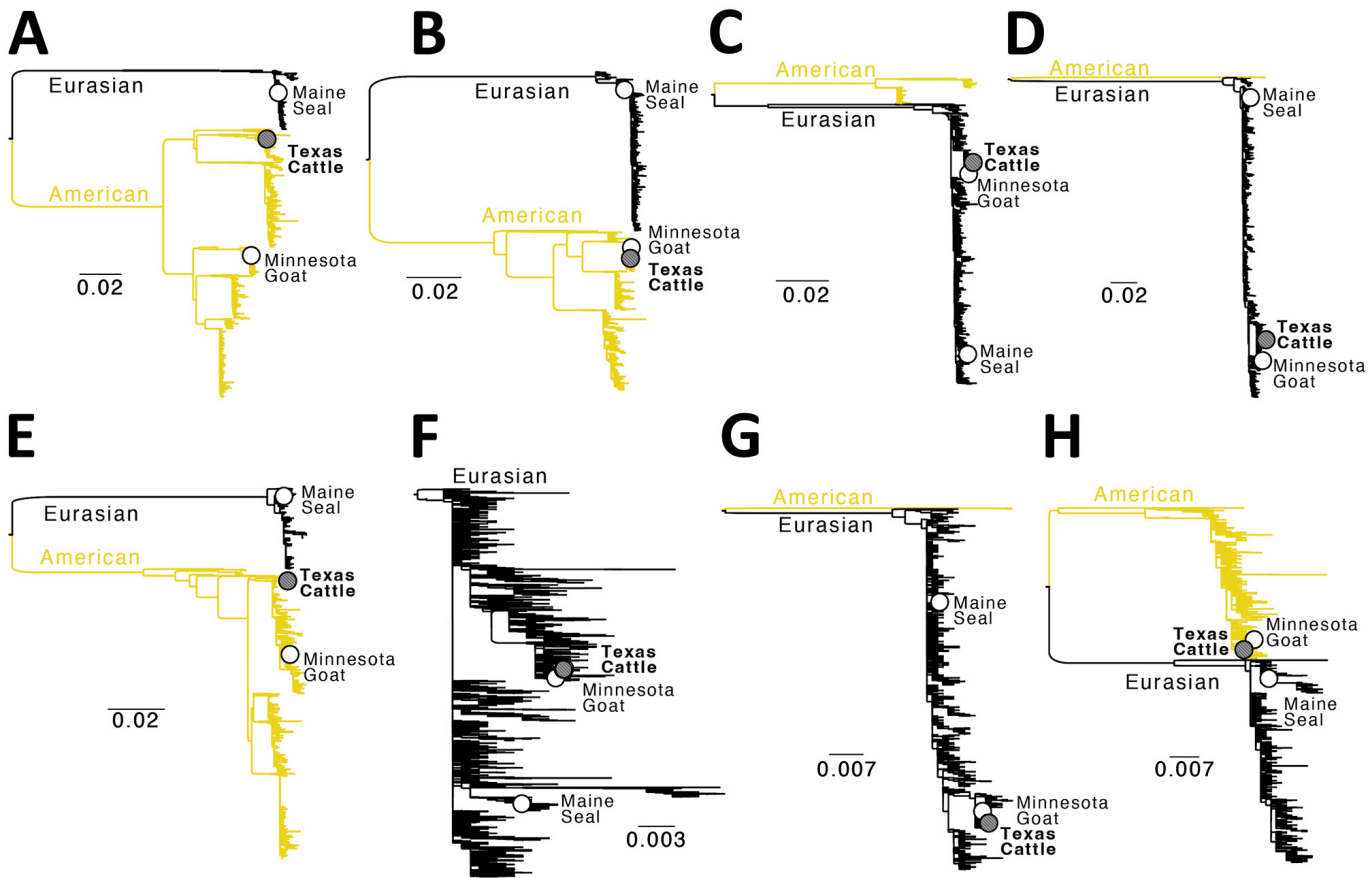
Phylogenetic trees for 8 genome segments in study of avian influenza A(H5N1) virus among dairy cattle, Texas, USA. Maximum-likelihood phylogenetic trees inferred for each of the 8 segments of the influenza A virus genome, including A/cattle/Texas/56283/2024(H5N1) isolated in this study (positioned in the Texas Cattle clade defined in Figure 1) and 3516–3644 H5N1 sequences (depending on the segment) from North America and South America, collected December 21, 2021–March 28, 2024, that were downloaded from GISAID (https://www.gisaid.org) on April 10, 2024. A) Polymerase basic 1; B) polymerase basic 2; C) polymerase acidic; D) hemagglutinin; E) nucleoprotein; F) neuraminidase; G) matrix; H) nonstructural. Outbreaks in Maine harbor seals and gray seals (June 2022), Minnesota goats (March 2024), and Texas cattle/humans (March 2024) are labeled. The American and Eurasian avian influenza lineage are labeled. All branch lengths drawn to scale. Scale bars indicate number of substitutions per site.

Multiple cattle swab specimens demonstrated molecular evidence of H5 avian influenza A virus (Appendix 1 Table 1). Of the first 20 cattle swab samples received, none had evidence of adenovirus, coronavirus, enterovirus, or influenza D. Of those first 20 specimens, 3 (2 healthy cows, 1 sick cow) demonstrated molecular evidence of a Paramyxoviridae or Pneumoviridae virus (Appendix 1 Table 1**)**. Multiple swab cultures in MDBK, Vero E6, and MDCK cells also had molecular evidence of H5 avian influenza A virus. Molecular study of the HA cleavage site demonstrated that those viruses were highly pathogenic. Next-generation sequencing (NGS) corroborated these findings; 1 cultured cattle nasal swab specimen yielded a complete genome A/cattle/Texas/56283/2024 (H5N1) (GenBank accession nos. PP600140–7 for the 8 viral segments), confirmed to be HPAI and of clade 2.3.4.4b. We performed phylogenetic comparisons of related viruses in GenBank and GISAID (https://www.gisaid.org) for the entire genome (Figure 1) and the virus’s 8 gene segments (Figure 2), which documented similarity to 13 other viruses in the Texas epizootic clade. A/cattle/Texas/56283/2024 (H5N1) had several novel mutations in comparison to related viruses (Table). One mutation (PB2-M631L) increases the capability of H5N1 to replicate in human cells by enhancing the polymerase activity of the viruses in human cells. Pathogenicity studies in animal models will be necessary to better understand such viruses. NGS analyses suggested that the sick cow in which a nasal swab specimen tested positive for Paramyxoviridae or Pneumoviridae virus (cattle identification [ID] 49869) (Appendix 1 Table 1) had a bovine viral diarrhea virus (BVDV), indicating a possible cause of illness. 

**Table:** Identified mutations, by gene segment, in avian influenza A(H5N1) strain A/cattle/Texas/56283/2024 (H5N1) detected in cattle on a dairy farm, Texas, USA*

Mutation region	All mutations	Red mutation	Orange mutations	Reference strain
HA	K3N, G16S, N110S, L120M, L131Q, T139P, T156A, Q185R, V194I, A201E, T211I, V226A, N252D, E284G, M285V, I298V, K492E, I526V, V538A, I547M, V548M		N110S, L131Q, T139P, V226A	A/Sichuan/26221/2014 (H5N6)
M1	N82S, N85S, N87T, T140A, F144L, M165I, V166A, A200V, A227T, K230R, M248L			A/duck/Guangdong/E1/2012 (H10N8)
M2	R12K, K18N, V27A, I51V, R61G, D88N	V27A		A/mallard/Astrakhan/263/1982 (H14N5)
NA	I8T, V17I, I20V, H44Y, A46P, V67I, N71S, T76A, K78Q, A81T, V99I, H100Y, H155Y, T188I, M258I, L269M, E287D, T289M, V321I, G336S, V338M, S339P, P340S, N366S, G382E, A395E, I418M, S434N, D460G			A/goose/Guangdong/1/1996 (H5N1)
NP	Y52H, S482N			A/chicken/BCFAV8//2014 (H5N2)
NS1	S7L, R21Q, S87P, C116S, D139N, A223E, V226I		A223E, V226I	A/duck/Guangdong/E1/2012 (H10N8)
NS2	E67G		E67G	A/quail/Italy/1117/1965 (H10N8)
PA	A36T, I61M, T85A, K113R, L219I, S277P, A404S, M441V, K497R, Y535H, I543L, S558L, T608S		A36T, A404S	A/Netherlands/219/2003 (H7N7)
PB1	T59S, E75D, I114V, I171V, M179I, S384T, V401A, A587P			A/Singapore/1/1957 (H2N2)
PB2	T58A, V109I, V139I, E362G, K389R, D441N, V478I, V495I, M631L, V649I, M676A			A/duck/Guangdong/E1/2012 (H10N8)

*As listed in FluSurver (http://flusurver.bii.a-star.edu.sg), red mutations alter viral virulence, cause strong drug resistance, or reverse the effects of the premature STOP codon. These have an assigned warning level of 3 (most significant); Orange mutations are those at drug binding sites or sites that alter host-cell specificity. These have a warning level 2 (significant). In addition, mutations at sites known to result in antigenic shifts or cause mild drug resistance are in this group. HA, hemagglutinin; M, matrix; NA, neuraminidase; NP, nucleoprotein; NS, nonstructural; PA, polymerase acidic; PB, polymerase basic.

## Conclusions

This preliminary study of a single Texas dairy farm affected by what is now a multistate epizootic of HPAI H5N1 documents several key observations. H5N1 virus detections were made solely in the sick cows without apparent co-infecting viruses (5 other viral families examined). HPAI H5N1 virus was more prevalent among nasal swab samples than rectal swab samples, supporting the notion that the respiratory tract of cattle could be involved in cow-to-cow transmission.

Although 1 sick cow (cattle ID 49869; Appendix 1 Table 1) was found by NGS to have evidence of a BVDV in its nasal swab specimen, 2 other healthy cows also had panspecies evidence of such a virus (cattle IDs 74061 and 54972; Appendix 1 Table 1); however, we did not perform NGS on their specimens. BVDVs are frequently associated with mild respiratory disease on cattle farms; because this farm routinely administers a vaccine with live BVDV components, we doubt that the BVDV explains the unusual illness seen in this farm’s dairy cattle herd.

The complete genome of the H5N1 virus isolated from 1 sick cow’s nasal swab specimen suggests that this H5N1 strain is very similar to the H5N1 strains characterized from dead birds, other cattle (*14*), and 1 cattle worker (*15*). The high genetic similarity of A/cattle/Texas/56283/2024 (H5N1) and other avian, human, and cattle strains in the Texas clade suggests a single interconnected multispecies outbreak in Texas, the precise directions of transmission still to be determined.

A limitation of our study is that we only examined specimens sent to us. We did not collect milk, study animal workers, or collect environmental specimens, nor did we immediately visit the farm for a comprehensive outbreak investigation. However, many barriers to performing a more traditional outbreak investigation on HPAI-infected farms currently exist.

The ongoing multispecies HPAI H5N1 outbreak involving birds, cattle, goats, alpacas, humans, cats, and other species epitomizes why interdisciplinary cooperation under a One Health framework is required. If we wish to resolve complex problems such as this epizootic, finding ways to assure farm owners that necessary epidemiological investigations will not harm their businesses will be imperative.

Appendix 1 Additional information about avian influenza A(H5N1) virus among dairy cattle, Texas, USA.

Appendix 2 Additional data from study of avian influenza A(H5N1) virus among dairy cattle, Texas, USA.
