# Supplementary material for: Avian Influenza A(H5N1) Virus among Dairy Cattle, Texas, USA
Source: Emerg Infect Dis. 2024 Jul;30(7):1425–9. doi: 10.3201/eid3007.240717 (PMC11210641; doi:10.3201/eid3007.240717)
Supplement: Appendix 1 — Additional information about avian influenza A(H5N1) virus among dairy cattle, Texas, USA. [file 24-0717-Techapp-s1.pdf]

Article DOI: https://doi.org/10.3201/eid3007.240717

EID cannot ensure accessibility for Supplemental Materials supplied by authors. Readers who have difficulty accessing supplementary content should contact the authors for assistance.

Avian Influenza A(H5N1) Virus Among Dairy Cattle, Texas, USA

Appendix 1

Materials and Methods

Molecular screening

Nucleic Acid Extractions

Two methods were used for viral RNA extraction; one from 140 µl aliquots of nasal swab samples using the QIAamp Viral RNA Mini Kit (Qiagen, Valencia, CA) according to manufacturer's instructions on the QIAcube automated extraction system (Qiagen), and second, 350 µl aliquots of bovine nasal swab samples were transferred to the UTMB's Assay Development Service Division (ADSD). Samples were deposited into individual wells of 96 deep-well processing plates (Roche Applied Science, Indianapolis, IN). Nucleic acids were extracted in high-throughput fashion using a Magna Pure 96 instrument employing large-volume Cellular RNA extraction kits (Roche) according to the manufacturer's protocol producing 50 µl of purified RNA. A portion of the RNA was immediately reverse transcribed to cDNA (iScript, Bio-Rad, Hercules, CA) and the remaining material was aliquoted and archived at −80°C. In addition, DNA was extracted using the QIAamp DNA Mini Kit (Qiagen) as recommended.

Influenza A and D RNA Reverse Transcription Polymerase Chain Reaction

Influenzas A and D viruses were among our initial diagnostic considerations as possible etiologies of the outbreak. To confirm our suspicion, real-time reverse transcription polymerase chain reaction (qRT-PCR) targeting the *matrix (M)* gene of influenza A virus (*1*) and the *nucleoprotein* (*NP)* gene of influenza D virus (*2*) was carried out on the RNA extract. Following positive Influenza A results from our initial testing, further confirmation for influenza detection

was performed by two additional assays at UTMB's Assay Development Service Division (ADSD). For this assay, a pan-influenza virus assay that targets the polymerase (*PB1)* gene and one for the influenza A virus polymerase (*PA)* gene were used (*3*) (Appendix 1 Table 1). RNA was first reverse transcribed to form cDNA using an iScript synthesis kit (Bio-Rad) in 10 µl reactions that were assembled in 96 well PCR plates (Thermofisher Scientific, Waltham, MA) by mixing of 2 µl iScript reaction mix, 0.5 µl reverse transcription and 7.5 µl of extracted RNA. Reverse transcription was completed using a Bio-Rad C1000 thermocycler using the protocol: 1) 25 C, 5.0 minutes, 2) 42 C, 30 minutes, 3) 85 C, 5.0 minutes, 4) 25 C, 5.0 minutes, 5) indefinite hold at 4 C. Synthesized cDNA was then used for PCR.

To evaluate *PB1* and *PA* genes by qRT-PCR, starting quantity values were extrapolated from standard curves of a plasmid harboring the PCR target generated in parallel for each run. Water only samples were included as contamination controls. Each 25 µl PCR reaction contained 12.5 µl of iQ SYBR® Supermix (Bio-Rad) 1 µl of both forward and reverse primers (5 µM), 3 µl cDNA and 7.5 µl total organic carbon-free water (Thermofisher Scientific). qPCR was completed in an OPUS Real-Time PCR system thermocycler (Bio-Rad) using the following parameters for influenza genes: Cycle 1), 95 C, 3.0 minutes, Cycle 2), Step 1. 95 C, 30 seconds, Step 2. 60 C, 30 seconds, Step 3. 72 C, 30 seconds repeat Cycle 2, 39x, Melt-temperature gradient 78 C to 83 C, 0.2 C temperature increments combined with 5 second reads. Fluorescent signal data was collected at the end of each annealing/extension and melt-temperature gradient step. A Melt-curve temperature gradient was used to match experimental samples to the control plasmid.

To determine the influenza A virus subtype, we employed hemagglutinin (*HA*) and neuraminidase (*NA*) RT-PCR assays for the detection of all types of influenza A viruses based on Hoffmann et al.'s protocol (*5*), a *HA* cleavage site RT-PCR assay (*9*), and a qRT-PCR screening assay for H5 (*1*).

Pan-species assays

Per our previous work (*10*,*11*) we used pan-species assays to study viral families known to cause respiratory epidemics. RNA was screened via pan-species conventional RT-PCR assays for *Coronaviridae* (*6*) Paramyxoviridae/Pneumoviridae (*8*), and *Enteroviridae* (*4*) *viruses.* DNA was screened for *Adenoviridae* (*7*) (Appendix 1 Table 1).

RT-PCR or PCR amplicons were analyzed by electrophoresis on a 1% agarose gel. Amplicons of expected targeted molecular weights from various PCR screenings were sent to Azenta Life Sciences (South Plainfield, NJ) for Sanger sequencing.

Cell culture

Attempts were made to isolate virus from samples found to be positive for influenza A virus through RT-PCR screening in Madin–Darby bovine kidney (MDBK) (ATCC, cat no. CCL-22), Vero E6 (ATCC, Manassa, VA) and Madin-Darby canine kidney (MDCK) (ATCC, cat no. CRL-CCL34) cell lines. All cell lines were grown in Minimal Essential Medium with glutamine (MEM, ThermoFisher Scientific, cat no. 11095080 Waltham, MA) supplemented with 10% fetal bovine serum (FBS, ThermoFisher Scientific cat no. 26140–079) and antibiotic-antimicrobic (ThermoFisher Scientific, cat no. 15240–062).

Infection of cells with samples

At 80%–90% confluency in 6-wells plates, the cells were washed with phosphate buffered saline (PBS, ThermoFisher Scientific). Infection medium was prepared with MEM supplemented with 0.1% FBS (ThermoFisher Scientific cat no. 26140–079) and 2 µg/mL TPCK-Trypsin (Sigma, cat no. 4352157–1KT). Prior to infecting the cells, a set of samples were treated with penicillin-streptomycin (5,000 U/mL) (1:1) and another was filtered using a 0.45 uM pore-size filter (Millipore Sigma Millex-HV Sterile Syringe Filter Unit, PVDF, 0.45 µm, Millipore, cat no. SLHV033RS). The treated and filtered samples (200 µl with 800 µl infection media) were inoculated onto the cell monolayers. The infected cells were incubated for 1 hour at 37°C in a 5% CO_2 environment for absorption. After the incubation, the cells were washed with PBS and 3mL infection media added to the cell monolayers and incubated at 37°C in a 5% CO_2. The cells were observed daily for CPE for 7 days before harvesting.

For molecular analyses of cell cultures displaying cytopathic effect (CPE), virus cultures treated with TRizol LS Reagent (Invitrogen, Waltham, MA) under BSL3 conditions before being moved into BSL2E where they underwent RNA extraction following the manufacturer's recommendations and were then stored at −80°C.

Next generation sequencing

To evaluate RNA quality, the bovine glyceraldehyde-3-phosphate dehydrogenase (GAPDH) housekeeping gene was quantified by qRT-PCR using species conserved primers for

each sample before metagenomics next generation sequencing (mNGS) (*12*). NEBNext Ultra II RNA Library Prep Kit for Illumina workflow was used for preparation of mNGS sequencing libraries according to manufactures recommendations. Sequencing was performed on the Illumina NextSeq550 platform paired end 75bp with an estimated 20 million reads per sample output. cDNA libraries were generated using a NEBNext Ultra II RNA Library Prep Kit for Illumina workflow (New England Biolabs Ipswich, MA) and the libraries for the five samples were pooled and sequenced on an Illumina NextSeq550 platform (Illumina, Inc., San Diego, CA). Mid Output flowcell for paired end 75 bp read length.

Bioinformatic Analysis

Sanger Sequencing Analysis

To perform rapid sequencing comparison, the Basic Local Alignment Search Tool (BLAST) available on the National Center for Biotechnology Information (NCBI) platform was used.

Additionally, the EMBOSS sixpack tool was employed for the preparation of the six-frame translation of the nucleotide sequences. For analysis purposes, translations with a minimum number of Open Reading Frames (ORFs) were selected.

Positive samples were differentiated from HPAI or low pathogenic avian influenza (LPAI) virus subtypes by an RT-PCR targeting the *HA* cleavage site with genome segment followed by sequencing of the resulting correct-sized amplicons.

Phylogenetic analyses for *HA* cleavage site of influenza A virus (IAV) were conducted using Geneious Prime software v2023.2.1 (Boston, MA). The Tamura-Nei model was employed to calculate genetic distance values.

Metagenomics Next Generation Sequence Analysis

Bovine host sequences (GCF_002263795.3) were removed using bowtie2 (*13*). De novo assembly of the remaining untrimmed paired-end reads was performed using abyss v2.3.7 using a range of kmer size from 19 to 41 (*14*). The resulting contigs were clustered using cd-hit v4.8.1 (*15*). BLASTX searches of the resulting contigs against a custom virus protein database and BLASTN searches of the resulting contigs against NCBI nt database, revealed an avian influenza

A genome with an average coverage of 105,400 reads/nucleotide for specimen UTMB number 11. The other specimen did not have enough reads to de novo assemble any contigs.

To construct a maximum likelihood phylogenetic tree for *HA* and *NA* segments, 100 sequences with the *HA* and *NA* sequences from this study via BLAST search were downloaded from NCBI GenBank. Sequences were filtered for completeness before Multiple sequence alignments using MAFFT (*16*). Midpoint-rooted maximum-likelihood phylogeny was estimated using IQTRee version 1.6.12 (*17*) with a GTR nucleotide substitution model and ultrafast bootstrapping. Figtree v1.4.4 (http://tree.bio.ed.ac.uk/software/figtree/) and the interactive tree of life (Itol) (*18*) were used to visualize and annotate trees.

Results

Molecular screening

The 40 swab specimens from 30 cows (24 ill) yielded 7 nasal swab specimens (all from ill cows) with molecular evidence of influenza A (Ct values ranging 34.7 to 38.2, Appendix 1 Table 2). Among the influenza A positives, 2 of 7 had indications of H5 subtype with Ct values of 35.3 and 39.1. None of the 20 first cattle swab samples received had evidence of adenovirus, coronavirus, enterovirus, or influenza D. Three (2 healthy cows, 1 sick cow) of the first 20 cattle swab specimens had molecular evidence of a *Paramyxoviridae/Pneumoviridae* virus but the Sanger sequence work from the amplicons could not be interpreted.

Next, we studied nasal swabs from 7 cattle (6 from sick cows with evidence of influenza A and 1 from a healthy cow with no evidence) for *HA* and *NA* gene characteristics by two methods: 1) Conventional RT-PCR with universal primers targeting the *HA* gene per WHO guidelines (*1*) and 2) UTMB's Assay Development Service Division's (ADSD) pan-influenza and influenza A qRT-PCR surveillance primers (*3*). The WHO *HA* assay produced amplicon products of the correct molecular weight with Sanger sequences from two samples showing 99.4% and 99.5% identity to known H5N1 sequences on NCBI.

UTMB's ADSD screening yielded positives for three of the six nasal swabs from ill cows, with evidence of influenza A (5.02E+02 - 2.11E+03 viral copies/ml) with 2 of the 3 samples concordant for positivity by influenza A surveillance by the 2nd set of primers. The nasal swab from the healthy cow was negative by both influenza qRT-PCR assays.

Cell culture

On March 26th we inoculated UTMB ID samples 9, 11, 13, 14, 20 and a negative control onto MDBK and Vero E6 cells. On March 28th, 2 days after inoculating MDBK and Vero E6 cells, we inoculated UTMB ID samples 8, 11, 13, 14, 20 and a negative control onto MDCK cells.

MDBK cell line

On March 30th, after 5 days of incubation, we harvested media from cells (1st harvest) and replaced it with fresh maintenance media. RNA was extracted from the harvest and the five samples were examined with qRT-PCR for influenza A and H5. All assays were negative. On April 2nd, after 7 days of incubation, we again harvested media from the cells (2nd harvest), inactivated the media with TRizol LS, extracted RNA from the media, and again examined the five specimens with qRT-PCR for influenza A and H5. All 5 samples had evidence of influenza A with Ct values ranging from 32.2 to 35.5 (Appendix 1 Table 3). These same five samples had H5 Ct values ranging from 36.5 to 41.5. All five samples were examined by RT-PCR for the *HA* cleavage site and all samples had a band of the correct molecular weight. Sanger sequencing confirmed HPAIV. Once high pathogenicity was suspected, specimens were transferred to BSL3E.

Vero E6 cell line

On March 30, after 5 days of incubation we harvested spent media from cells (1st harvest) and replaced with fresh infection media. RNA was extracted from the harvest and examined the five specimens with qRT-PCR for influenza A and H5. All assays were negative. On April 2, after 7 days of incubation, we again harvested cells (2nd harvest), inactivated with TRizol LS, extracted RNA, and again examined the five specimens with qRT-PCR for influenza A and H5. Three samples had evidence of influenza A with Ct values ranging from 36.3 to 37.8 (Appendix 1 Table 3). These same three samples had H5 Ct values ranging from 41.2 to 45.2. All five samples were examined with RT-PCR for the *HA* cleavage site and one sample had a band of the correct molecular weight. We did not send this amplicon for sequencing. Once high pathogenicity was suspected, specimens were transferred to BSL3E.

MDCK cell line

On April 2, we harvested spent media from the MDCK cells after 5 days of incubation (1st harvest), inactivated the specimens with TRizol LS, extracted RNA, and examined the five specimens with qRT-PCR for influenza A and H5. Four of the five samples had evidence of influenza A with Ct values ranging from 13.6 to 36.7 (Appendix 1 Table 3). Three of the five samples had H5 Ct values ranging from 20.2 to 41.4. All five samples were examined with RT-PCR for the *HA* cleavage site and 2 of 5 samples had a band of the correct molecular weight. Amplicons from these two samples were sent for Sanger sequencing. Once high pathogenicity was suspected, specimens were transferred to BSL3E.

We received good Sanger sequencing results from 5 cattle swab samples grown in MDBK cells and 2 samples grown in MDCK cells. Sanger sequencing results for the 5 swab samples demonstrated the presence of multiple basic amino acid (PLREKRRKRGLF) at the HA cleavage site indicating that bovine strains were HPAIV H5N1 viruses belonging to clade 2.3.4.4b. (Appendix 1 Figure 1). Phylogenetic analyses demonstrated our five Sanger sequences were closely related to the A/Texas/37/2024(H5N1) (GenBank accession number PP577943.1).

Next generation sequencing

RNA extracts from original swab specimens from four sick cows were selected for next generation sequencing (NGS): three had molecular evidence of influenza A virus and one had molecular evidence of paramyxoviridae/pneumoviridae virus. A fifth specimens was also sent for NGS: RNA with low Ct value harvested from MDCK cells (same cow as one of the original nasal swabs). The libraries for the five samples were pooled and sequenced. Upon completion of the sequencing run, an estimated total of 166 million read pairs were obtained for the five samples (specimen UTMB number 11 with 25 million read pairs; specimen UTMB number 16, 34 million read pairs; specimen UTMB number 3, 34 million read pairs; specimen UTMB number 5, 34 million read pairs; and specimen UTMB number 6, 32 million read pairs). Bovine host sequences (GCF_002263795.3) were removed using bowtie2 (*12*). De novo assembly of the remaining untrimmed paired-end reads was performed using abyss v2.3.7 using a range of kmer size from 19 to 41 (*14*) The resulting contigs were clustered using cd-hit v4.8.1 (*15*). BLASTX searches of the resulting contigs against a custom virus protein database and BLASTN searches of the resulting contigs against NCBI nt database, revealed an avian influenza A genome with an

average coverage of 105,400 reads/nucleotide for specimen UTMB number 11. The other specimen did not have enough reads to de novo assemble any contigs.

All qRT-PCR positive samples sequenced had reads for influenza A, with the highest reads per million of over 950,000 for the sample grown on MDCK. In one of the sick cow samples subjected to mNGS, pestivirus, the etiologic agent of bovine viral diarrhea virus (BVDV) reads, were detected, with 20% genome coverage following genome assembly.

The entire genome of influenza A from the nasal swab specimen UTMB number 11 was assembled using metagenomic methods (GenBank accession number for the eight viral segments PP600140- PP600147). Gene segment coverage was good (Appendix 1 Figure 2). Phylogenetic comparisons were made of related viruses in GenBank/GISAID for the entire genome (Figure 1 in main article), and the *HA* (Appendix 1 Figure 3) and *NA* (Appendix 1 Figure 4) gene segments.

Phylogenetic Analyses

Our genome sequence, A/cattle/Texas/56283/2024 (H5N1), was confirmed to be HPAIV belonging to clade 2.3.4.4b of the H5N1 subtype. The virus is positioned within the same clade as a human sample from Texas recently reported by CDC (*19*). Including our sample, this Texas clade now includes 13 HPAI H5N1 viruses collected during March 2024 from dairy cattle (n = 8), blackbird (n = 2), common grackle (n = 2), and human (A/Texas/37/2024/H5N1), which were downloaded from GISAID on April 10, 2024 (Figure 1 in main article; Appendix 2, https://wwwnc.cdc.gov/EID/article/30/7/24-0717-App2.xlsx). All 13 viruses in the Texas clade have the same 4:4 reassortant genotype (genotype B3.13), with PB2, PB1, NP, and NS segments from the American avian lineage and PA, HA, NA, and MP segments from the Eurasian avian lineage (Figure 2 in main article). The close genetic relatedness (>99.7%) of the genomes of the 13 Texas H5N1 viruses suggests they belong to the same multispecies outbreak involving avian, human, and cattle hosts. The genetic similarity of A/cattle/Texas/56283/2024 (H5N1) and other H5N1 viruses recently obtained from Texas cattle points to a common source of infection or farm-to-farm spread. Three H5N1 viruses obtained on March 8, 2024 from an outbreak in goats in Minnesota have the same 4:4 reassortant genotype, but are positioned in a different part of the phylogenetic tree, not closely related to the Texas cattle viruses (most visible on the PB2 segment, Figure 2 in main article, raw tree files for all segments are available at

https://github.com/mostmarmot/TexasH5N1). The Minnesota goat viruses do not appear to have a connection to the Texas cattle outbreak and are likely an independent virus introduction from birds to goats.

Mutations

Across the genome we identified several differences between A/cattle/Texas/56283/2024 (H5N1) and the other related genomes from the Texas clade (Appendix 1 Figure 5). The V27A substitution in the M2 gene is a relatively rare mutation is associated with strong to mild adamantane resistance (*20*). However, it has been detected in H5 HPAI GS/Gd lineage viruses (*21*). Resistance to adamantane drugs is relatively rare among avian influenza isolates (*22*). Fortunately, adamantane drugs are no longer recommended for treatment of humans with influenza A or B infection. Other mutations associated with virulence were identified in the *PB2* (V495I, M676A, and M631L)*NS1* (A223E) genes (Table in main article). In particular, the M63IL mutation enhances viral polymerase activity, thereby increasing the ability of the virus to replicate in mammalian hosts (*23*). N110S and V226A mutations in the *HA* gene are associated with host specificity shift while L131Q and T156A are antigenic drift/escape mutations (*21*).

References

1. World Health Organization. Manual for the laboratory diagnosis and virological surveillance of influenza [cited 2024 Mar 18]. https://www.who.int/publications/i/item/manual-for-the-laboratory-diagnosis-and-virological-surveillance-of-influenza

2. Henritzi D, Hoffmann B, Wacheck S, Pesch S, Herrler G, Beer M, et al. A newly developed tetraplex real-time RT-PCR for simultaneous screening of influenza virus types A, B, C and D. Influenza Other Respir Viruses. 2019;13:71–82. PubMed https://doi.org/10.1111/irv.12613

3. Sampath R, Russell KL, Massire C, Eshoo MW, Harpin V, Blyn LB, et al. Global surveillance of emerging Influenza virus genotypes by mass spectrometry. PLoS One. 2007;2:e489. PubMed https://doi.org/10.1371/journal.pone.0000489

4. Oberste MS, Feeroz MM, Maher K, Nix WA, Engel GA, Hasan KM, et al. Characterizing the picornavirus landscape among synanthropic nonhuman primates in Bangladesh, 2007 to 2008. J Virol. 2013;87:558–71. PubMed https://doi.org/10.1128/JVI.00837-12

5. Hoffmann E, Stech J, Guan Y, Webster RG, Perez DR. Universal primer set for the full-length amplification of all influenza A viruses. Arch Virol. 2001;146:2275–89. PubMed https://doi.org/10.1007/s007050170002

6. Xiu L, Binder RA, Alarja NA, Kochek K, Coleman KK, Than ST, et al. A RT-PCR assay for the detection of coronaviruses from four genera. J Clin Virol. 2020;128:104391. PubMed https://doi.org/10.1016/j.jcv.2020.104391

7. Wellehan JF, Johnson AJ, Harrach B, Benkö M, Pessier AP, Johnson CM, et al. Detection and analysis of six lizard adenoviruses by consensus primer PCR provides further evidence of a reptilian origin for the atadenoviruses. J Virol. 2004;78:13366–9. PubMed https://doi.org/10.1128/JVI.78.23.13366-13369.2004

8. Tong S, Chern SW, Li Y, Pallansch MA, Anderson LJ. Sensitive and broadly reactive reverse transcription-PCR assays to detect novel paramyxoviruses. J Clin Microbiol. 2008;46:2652–8. PubMed https://doi.org/10.1128/JCM.00192-08

9. Slomka MJ, Coward VJ, Banks J, Löndt BZ, Brown IH, Voermans J, et al. Identification of sensitive and specific avian influenza polymerase chain reaction methods through blind ring trials organized in the European Union. Avian Dis. 2007;51(Suppl):227–34. PubMed https://doi.org/10.1637/7674-063006R1.1

10. Bailey ES, Fieldhouse JK, Choi JY, Gray GC. A mini review of the zoonotic threat potential of influenza viruses, coronaviruses, adenoviruses, and enteroviruses. Front Public Health. 2018;6:104. PubMed https://doi.org/10.3389/fpubh.2018.00104

11. Gray GC, Robie ER, Studstill CJ, Nunn CL. Mitigating future respiratory virus pandemics: new threats and approaches to consider. Viruses. 2021;13:637. PubMed https://doi.org/10.3390/v13040637

12. Urban RJ, Pyles RB, Stewart CJ, Ajami N, Randolph KM, Durham WJ, et al. Altered fecal microbiome years after traumatic brain injury. J Neurotrauma. 2020;37:1037–51. PubMed https://doi.org/10.1089/neu.2019.6688

13. Langmead B, Salzberg SL. Fast gapped-read alignment with Bowtie 2. Nat Methods. 2012;9:357–9. PubMed https://doi.org/10.1038/nmeth.1923

14. Jackman SD, Vandervalk BP, Mohamadi H, Chu J, Yeo S, Hammond SA, et al. ABySS 2.0: resource-efficient assembly of large genomes using a Bloom filter. Genome Res. 2017;27:768–77. PubMed https://doi.org/10.1101/gr.214346.116

15. Li W, Godzik A. Cd-hit: a fast program for clustering and comparing large sets of protein or nucleotide sequences. Bioinformatics. 2006;22:1658–9. PubMed https://doi.org/10.1093/bioinformatics/btl158

16. Katoh K, Standley DM. MAFFT multiple sequence alignment software version 7: improvements in performance and usability. Mol Biol Evol. 2013;30:772–80. PubMed https://doi.org/10.1093/molbev/mst010

17. Trifinopoulos J, Nguyen LT, von Haeseler A, Minh BQ. W-IQ-TREE: a fast online phylogenetic tool for maximum likelihood analysis. Nucleic Acids Res. 2016;44(W1):W232-5. PubMed https://doi.org/10.1093/nar/gkw256

18. Letunic I, Bork P. Interactive Tree Of Life (iTOL) v5: an online tool for phylogenetic tree display and annotation. Nucleic Acids Res. 2021;49(W1):W293–6. PubMed https://doi.org/10.1093/nar/gkab301

19. Centers for Disease Control and Prevention. Technical update: summary analysis of genetic sequences of highly pathogenic avian influenza A(H5N1) viruses in Texas [cited 2024 Mar 18]. https://www.cdc.gov/flu/avianflu/spotlights/2023-2024/h5n1-analysis-texas.htm

20. Thomaston JL, Konstantinidi A, Liu L, Lambrinidis G, Tan J, Caffrey M, et al. X-ray crystal structures of the influenza M2 proton channel drug-resistant V27A mutant bound to a spiro-adamantyl amine inhibitor reveal the mechanism of adamantane resistance. Biochemistry. 2020;59:627–34. PubMed https://doi.org/10.1021/acs.biochem.9b00971

21. Kandeil A, Patton C, Jones JC, Jeevan T, Harrington WN, Trifkovic S, et al. Rapid evolution of A(H5N1) influenza viruses after intercontinental spread to North America. Nat Commun. 2023;14:3082. PubMed https://doi.org/10.1038/s41467-023-38415-7

22. He W, Zhang W, Yan H, Xu H, Xie Y, Wu Q, et al. Distribution and evolution of H1N1 influenza A viruses with adamantanes-resistant mutations worldwide from 1918 to 2019. J Med Virol. 2021;93:3473–83. PubMed https://doi.org/10.1002/jmv.26670

23. Centers for Disease Control and Prevention. Technical update: summary analysis of the genetic sequence of a highly pathogenic avian influenza A(H5N1) virus identified in a human in Michigan [cited 2024 May 24]. https://www.cdc.gov/flu/avianflu/spotlights/2023-2024/h5n1-technical-update-may-24-2024.html

Appendix 1 Table 1. Molecular assays used in this investigation*

Target	Primers	Probe	Reference	Comments
qRT-PCR Influenza A (*M* gene)	InfA-F: GACCRATCCTGTCACCTCTGA C<br><br>InfA-R: AGGGCATTYTGGACAAAKCGTCTA	InfA-P: 6FAM-ZEN-TGC AGT CCT CGC TCA CTG GGC ACG3IABkFQ	(*1*)	
qRT-PCR for influenza A *PB1* and *PA* genes(by ADSD) 5′-3′	PB1<br><br>F: TGTCCTGGAATGATGATGGGCATGTT<br>R: TCATCAGAGGATTGGAGTCCATCCC<br>PA<br>F: TGGGATTCCTTTCGTCAGTCCGA<br>R: TGGAGAAGTTCGGTGGGAGACTTTGGT	None	(*3*)	SYBR Green-Based Assay
qRT-PCR Influenza D (*NP* gene)	D_NP_F: CTTGAAAAGATTGCAAATGCAG<br><br>D_NP_R: GTTGGGTTTCAGTGCCATTC	D_NP_SO: HEX-CACTACATTTCCCAGCTGTTGACTCC-BHQ1	(*2*)	
qRT-PCR Influenza A H5	H5–1012Fw: TGGGTACCACCATAGCAATGAGCA<br><br>H5–1155Rv: AATTCCCTTCCAACGGCCTCAAAC	H5–1024P-P2: HEX–TGGGTACGCTGCAGACAAAGAATCCA–BHQ1	(*1*)	
qRT-PCR enterovirus	AN350: GGCCCCTGAATGCGGCTAATCC<br><br>AN351: GCGATTGTCACCATWAGCAGYCA	AN234_P: FAM–CCGACTACTTTGGGWGTCCGTGT- IBFQ	(*4*)	
RT-PCR Influenza A (universal full-length *HA* gene)	HA-Bm-HA-1: TATTCGTCTCAGGGAGCGAAAGCAGGTAC<br><br>Bm-NS-890R: ATATCGTCTCGTATTAGTAGAAACAAGGGTGTTTT	N/A	(*5*)	(expected size [bp]**)** 1778+29
RT-PCR Influenza A (universal full-length *NA* gene)	Ba-Na-1: TATTGGTCTCAGGGAGCAAAAGCAGGAGT<br><br>Ba-Na-1413R: ATATGGTCTCGTATTAGTAGAAACAAGGAGTTTTTT	N/A	(*5*)	(expected size [bp]**)** 1413+29
RT-PCR Influenza A (Universal HA2 fragment)	HA-1144: GGAATGATAGATGGNTGGTAYGG<br><br>NS-890R: AGTAGAAACAAGGGTGTTTT	N/A	(*5*)	(expected size [bp]**)** 700
RT-PCR pan-coronavirus	PCR_1<br><br>panCov_outF: CCAARTTYTAYGGHGGITGG<br>panCov_R: TGTTGIGARCARAAYTCATGIGG<br>PCR_2<br>panCov_InF: GGTTGGGAYTAYCCHAARTGTGA<br>panCov_R: TGTTGIGARCARAAYTCATGIGG	N/A	(*6*)	(expected size [bp]) of 599 −602
PCR pan-adenovirus	PCR_1<br><br>Pol-Fouter: TNMGNGGNGGNMGNTGYTAYCC<br>Pol-Router: GTDGCRAANSHNCCRTABARNGMRTT<br>PCR_2<br>Pol-Finner: GTNTWYGAYATHTGYGGHATGTAYGC<br>Pol_Rinner: CCANCCBCDRTTRTGNARNGTRA	N/A	(*7*)	(expected size [bp]) of 318 −324

Target	Primers	Probe	Reference	Comments
RT-PCR pan-paramyxovirus/pneumovirus	PCR_1<br>PAR-F1: GAAGGITATTGTCAIAARNTNTGGAC<br>PAR-R: GCTGAAGTTACIGGITCICCDATRTTNC<br>PCR_2<br>PAR-F2: GTTGCTTCAATGGTTCARGGNGAYAA<br>PAR-R: GCTGAAGTTACIGGITCICCDATRTTNC	N/A	(*8*)	
RT-PCR *HA* cleavage site	H5KHA-1: CCTCCAGARTATGCMTAYAAAATTGTC<br>H5 KHA-3: TACCAACCGTCTACCATKCCYTG	N/A	(*9*)	(expected size [bp]) 300–320

*N/A, not applicable; qRT-PCR, quantitative RT-PCR; RT-PCR, reverse transcription PCR.

Appendix 1 Table 2. Summary of field data and laboratory assay results from 40 dairy cattle swab specimens*

UTMB ID	Cattle ID	Type swab	Cattle age	Illness	Date collected on farm	FluA qRT-PCR (Ct). 1st run	FluA qRT-PCR (Ct), 2nd run	H5 qRT-PCR (Ct)	Pan-paramixo/pneumo RT-PCR	Pan-paramyxovirus/pneumovirus Sanger sequence results
1	73486	Nasal	2 y 6 m	No	3/21/2024	–	–	–	–	
2	74061	Nasal	2y 6m	No	3/21/2024	–	–	–	positive	No virus identified
3	54972	Nasal	3y 8m	No	3/21/2024	–	–	–	positive	No virus identified
4	75841	Nasal	2y 3m	No	3/21/2024	–	–	–	–	
5	583	Nasal	5y 1m	No	3/21/2024	–	–	–	–	
6	76748	Nasal	2y 3m	No	3/21/2024	–	–	–	–	
7	77580	Nasal	6y 10m	Yes	3/21/2024	–	–	–	–	
8	70234	Nasal	2y 11m	Yes	3/21/2024	37.9	–	39.1	–	
9	75526	Nasal	7y 5m	Yes	3/21/2024	37.0	36.7		–	
10	98727	Nasal	4y 9m	Yes	3/21/2024	–	–	–	–	
11	56283	Nasal	3y 4m	Yes	3/21/2024	34.7	–	–	–	
12	84179	Nasal	6y 7m	Yes	3/21/2024	–	–	–	–	
13	2135	Nasal	5y 4m	Yes	3/21/2024	37.9	37.5	–	–	
14	52612	Nasal	4y 6m	Yes	3/21/2024	37.4	38.2	–	–	
15	96953	Nasal	5y 1m	Yes	3/21/2024	–	–	–	–	
16	49869	Nasal	5y 3m	Yes	3/21/2024	–	–	–	positive	No virus identified
17	40625	Nasal	6y 2m	Yes	3/21/2024	–	–	–	–	
18	52622	Nasal	4y 6m	Yes	3/21/2024	–	–	–	–	
19	5335	Nasal	5y 3m	Yes	3/21/2024	–	–	–	–	
20	45112	Nasal	5y 10m	Yes	3/21/2024	35.5	36.0	–	–	
21	59068	Nasal	3y 1m	Yes	4/1/2024	–	–	–		
22	59068	Rectal			4/1/2024	–	–	–		
23	51726	Nasal	4y 10 m	Yes	4/1/2024	–	–	–		
24	51726	Rectal			4/1/2024	–	–	–		
25	6364	Nasal	4y 4m	Yes	4/1/2024	–	–	–		
26	6364	Rectal			4/1/2024	–	–	–		
27	82167	Nasal	7y 1m	Yes	4/1/2024	–	–	–		
28	82167	Rectal			4/1/2024	–	–	–		
29	79515	Nasal	6y 6m	Yes	4/1/2024	–	–	–		

UTMB ID	Cattle ID	Type swab	Cattle age	Illness	Date collected on farm	FluA qRT-PCR (Ct). 1st run	FluA qRT-PCR (Ct), 2nd run	H5 qRT-PCR (Ct)	Pan-paramixo/pneumo RT-PCR	Pan-paramyxovirus/pneumovirus Sanger sequence results
30	79515	Rectal			4/1/2024	–	–	–		
31	24755	Nasal	7y 10 m	Yes	4/1/2024	–	–	–		
32	24755	Rectal			4/1/2024	–	–	–		
33	75425	Nasal	2y 4 m	Yes	4/1/2024	–	–	–		
34	75425	Rectal			4/1/2024	–	–	–		
35	74117	Nasal	2y 6 m	Yes	4/1/2024	–	28.8	35.3		
36	74117	Rectal			4/1/2024	–	–	–		
37	58736	Nasal	9 y 5 m	Yes	4/1/2024	–	–	–		
38	58736	Rectal			4/1/2024	–	–	–		
39	79261	Nasal	2 y 1m	Yes	4/1/2024	–	–	–		
40	79261	Rectal			4/1/2024	–	–	–		

*The swabs were collected from 24 sick and 6 healthy dairy cattle from the farm on March 21xt or April 1st 2024. None of the 20 first cattle swab samples received had evidence of adenovirus, coronavirus, enterovirus or influenza D. qRT-PCR, quantitative RT-PCR; RT-PCR, reverse transcription PCR; UTMB, University of Texas Medical Branch.

Appendix 1 Table 3. Summary of laboratory assay results from six dairy cattle swab specimens during cell culture

		5 d after inoculation		7 d after inoculation				
Cattle ID	UTMB ID	FluA qRT-PCR (Ct)	H5 RT-PCR (Ct)	FluA qRT-PCR (Ct)	H5 RT-PCR (Ct)	HA0 motif by RT- PCR	Sanger sequencing performed?	HPAI/LPAI
MDBK cells								
75526	9	negative	negative	33.1	38.3	positive	yes	HPAI
56283	11	negative	negative	34.8	41.5	positive	yes	HPAI
2135	13	negative	negative	32.3	38.2	positive	yes	HPAI
52612	14	negative	negative	32.2	36.5	positive	yes	HPAI
45112	20	negative	negative	35.5	36.9	positive	yes	HPAI
Vero E6 cells								
75526	9	negative	negative	36.3	42.2	–	no	
56283	11	negative	negative	36.2	41.0	–	no	
2135	13	negative	negative	37.8	45.2	positive	no	
52612	14	negative	negative	–	–	–	no	
45112	20	negative	negative	–	–	–	no	
MDCK cells								
70234	8			–	–	–	no	
56283	11	13.6	20.2	–	–	positive	yes	HPAI
2135	13	35.9	41.4	–	–	–	no	
52612	14	34.5	38.0	–	–	positive	yes	HPAI
45112	20	36.7	–	–	–	–	no	

*HPAI, highly pathogenic avian influenza; LPAI, low pathogenic avian influenza; qRT-PCR, quantitative RT-PCR; RT-PCR, reverse transcription PCR; UTMB, University of Texas Medical Branch.

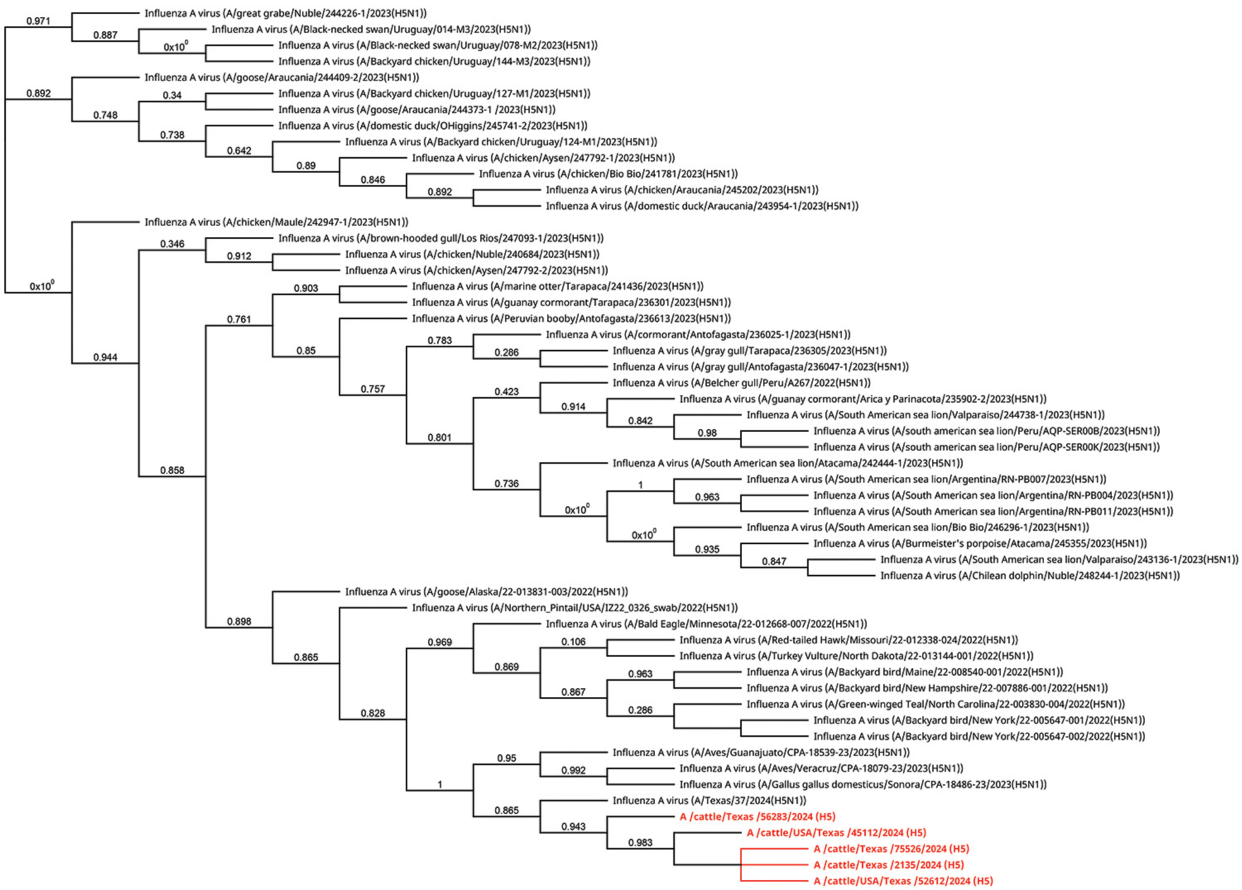

Appendix 1 Figure 1. A phylogenetic tree of the *HA* cleavage sites. These data are from five cattle nasal swab sample sequences that were in MDBK cells from this study (colored in red) compared to other related viruses in GenBank.

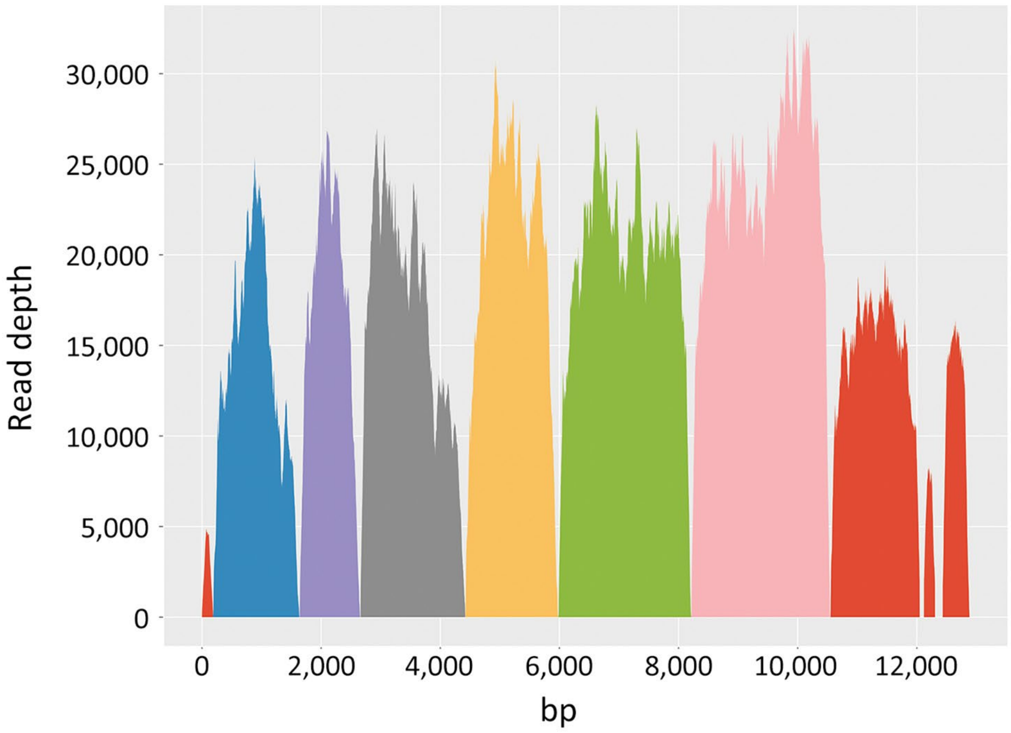

Appendix 1 Figure 2. Genome coverage plot for A/cattle/Texas/56283/2024(H5N1). This graphic shows the depth of coverage for the different segments of the virus. From left to right: First red (*NS1* and *NEP* genes), blue (*NA* gene), purple (*M1* and *M2* genes), gray (*HA* gene) yellow (*NP* gene), green (*PA* and *PA-X* genes), lilac (*PB1* and *PB1-F2* genes), last red (*PB2* gene).

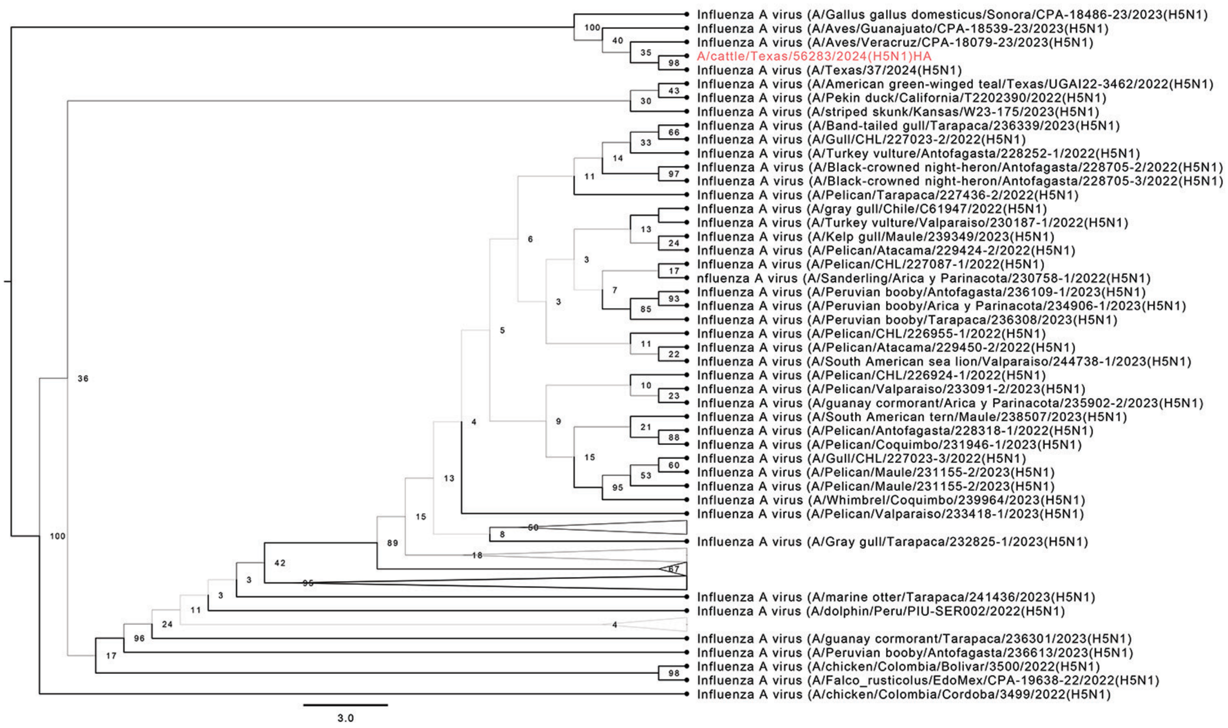

Appendix 1 Figure 3. A maximum likelihood phylogenetic tree of the hemagglutinin (*HA*) gene segment of A/cattle/Texas/56283/2024(H5N1)(red in color) compared to the *HA* gene segments from other related H5N1 viruses in GenBank.

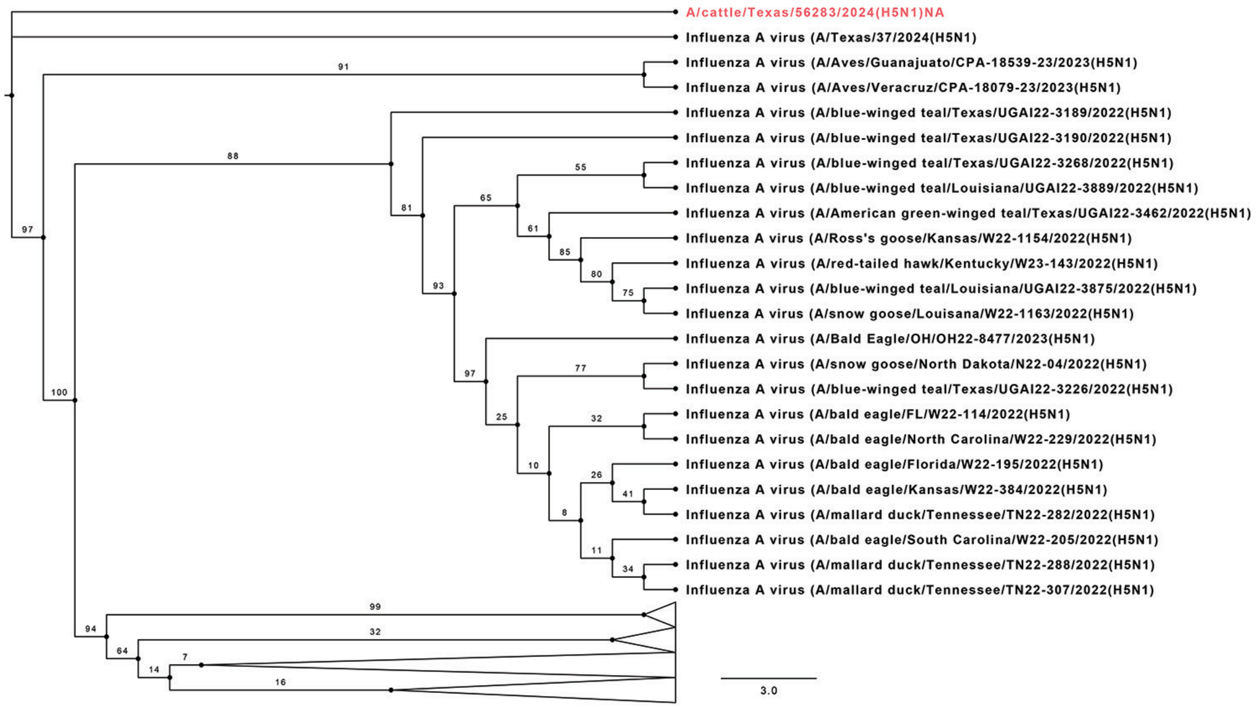

Appendix 1 Figure 4. A maximum likelihood phylogenetic tree of the neuraminidase (*NA*) gene segment between A/cattle/Texas/56283/2024(H5N1) isolated in this study (red in color) compared to the *NA* gene segments from other related H5N1 viruses in GenBank.

	PB2	PB2	PB1	NP	NS1	PA	PA	M2	NS1
	1242	1869	1889	762	651	36	404	27	21
A/blackbird/Texas/24-008354-001/2024(H5N1)	t	t	t	a	g	Ala	Ala	Val	Arg
A/blackbird/Texas/24-008357-001/2024(H5N1)	t	t	t	a	g	Ala	Ala	Val	Arg
A/common grackle/Texas/24-008356-001/2024(H5N1)	t	t	t	a	g	Ala	Ala	Val	Arg
A/common grackle/Texas/24-008356-003/2024(H5N1)	t	t	t	a	g	Ala	Ala	Val	Arg
A/dairy cattle/Texas/24-008749-001/2024(H5N1)	t	t	t	a	g	Ala	Ala	Val	Arg
A/dairy cattle/Texas/24-008749-002-v/2024(H5N1)	t	t	t	a	g	Ala	Ala	Val	Arg
A/dairy cattle/Texas/24-008749-003/2024(H5N1)	t	t	t	a	g	Ala	Ala	Val	Arg
A/dairy cattle/Texas/24-008749-004/2024(H5N1)	t	t	t	a	g	Ala	Ala	Val	Arg
A/dairy cattle/Texas/24-008749-005/2024(H5N1)	t	t	t	a	g	Ala	Ala	Val	Arg
A/dairy cattle/Texas/24-008749-006/2024(H5N1)	t	t	t	a	g	Ala	Ala	Val	Arg
A/dairy cattle/Texas/24-008749-007/2024(H5N1)	t	t	t	a	g	Ala	Ala	Val	Arg
A/Texas/37/2024(H5N1)	t	t	t	a	g	Ala	Ala	Val	Arg
**A/cattle/Texas/56283/2024(H5N1)**	**c**	**c**	**c**	**g**	**a**	**Thr**	**Ser**	**Ala**	**Gln**

Nucleotide substitutions (silent) | Amino acid substituions

Appendix 1 Figure 5. Mutations in A/cattle/USA/Texas/56283/2024(H5N1) across the genome. The genome sequence of A/cattle/Texas/56283/2024(H5N1) was compared against the 12 other sequences in the Texas clade (Figure 1 in main article). Five silent (not changing the amino acid encoded) nucleotide substitutions and four amino acid substitutions were identified.
